# Increased genetic gains in sheep, beef and dairy breeding programs from using female reproductive technologies combined with optimal contribution selection and genomic breeding values

**DOI:** 10.1186/s12711-015-0151-3

**Published:** 2015-09-14

**Authors:** Tom Granleese, Samuel A. Clark, Andrew A. Swan, Julius H. J. van der Werf

**Affiliations:** Sheep Cooperative Research Centre, Armidale, 2351 Australia; School of Environmental and Rural Science, University of New England, Armidale, 2351 Australia; Animal Genetics and Breeding Unit, Armidale, 2351 Australia

## Abstract

**Background:**

Female reproductive technologies such as multiple ovulation and embryo transfer (MOET) and juvenile in vitro embryo production and embryo transfer (JIVET) can boost rates of genetic gain but they can also increase rates of inbreeding. Inbreeding can be managed using the principles of optimal contribution selection (OCS), which maximizes genetic gain while placing a penalty on the rate of inbreeding. We evaluated the potential benefits and synergies that exist between genomic selection (GS) and reproductive technologies under OCS for sheep and cattle breeding programs.

**Methods:**

Various breeding program scenarios were simulated stochastically including: (1) a sheep breeding program for the selection of a single trait that could be measured either early or late in life; (2) a beef breeding program with an early or late trait; and (3) a dairy breeding program with a sex limited trait. OCS was applied using a range of penalties (severe to no penalty) on co-ancestry of selection candidates, with the possibility of using multiple ovulation and embryo transfer (MOET) and/or juvenile in vitro embryo production and embryo transfer (JIVET) for females. Each breeding program was simulated with and without genomic selection.

**Results:**

All breeding programs could be penalized to result in an inbreeding rate of 1 % increase per generation. The addition of MOET to artificial insemination or natural breeding (AI/N), without the use of GS yielded an extra 25 to 60 % genetic gain. The further addition of JIVET did not yield an extra genetic gain. When GS was used, MOET and MOET + JIVET programs increased rates of genetic gain by 38 to 76 % and 51 to 81 % compared to AI/N, respectively.

**Conclusions:**

Large increases in genetic gain were found across species when female reproductive technologies combined with genomic selection were applied and inbreeding was managed, especially for breeding programs that focus on the selection of traits measured late in life or that are sex-limited. Optimal contribution selection was an effective tool to optimally allocate different combinations of reproductive technologies. Applying a range of penalties to co-ancestry of selection candidates allows a comprehensive exploration of the inbreeding vs. genetic gain space.

## Background

Female reproductive technologies such as multiple ovulation and embryo transfer (MOET) and juvenile in vitro fertilization and embryo transfer (JIVET) can be used by breeders to accelerate genetic gain in livestock breeding programs. These technologies allow an increase of selection intensity placed on females and they can also reduce the optimal age at which animals are selected, and thus decrease generation intervals in the breeding program.

Prior to genomic selection (GS) [1], MOET breeding programs were reported to increase genetic gain in dairy breeding programs by up to 30 % by increasing the selection intensity of females [2]. Furthermore, if MOET was used and the age of selection candidates decreased, breeding schemes would increase genetic gain by another 9 % [3]. These gains were similar also in beef and sheep breeding programs, for which MOET was estimated to yield an extra 67 to 100 % of genetic gain for beef [4, 5] and 17 to 74 % for sheep [6–8]. However, all these studies agreed that MOET breeding programs would also increase annual rates of inbreeding by up to 110 % compared to traditional mating programs. Some of these increased rates of inbreeding could have even been underestimated due to reductions in genetic variance and to the assumption that the size of a family and family numbers were constant [9]. For example, the stochastic simulation of Villanueva et al. [10] showed that inbreeding rates increased by 17 % when there is variability between donors in embryo production compared to when embryo numbers are assumed fixed.

When JIVET became commercially viable in the 1990s, selection of donors for JIVET was often based on estimated breeding values (EBV) that had low accuracy and that were highly correlated among siblings due to the great emphasis put on common family information. In this scenario, it is difficult to realize high rates of genetic gain and maintain sustainable rates of inbreeding. Marker-assisted selection [11] and more recently genomic selection [1] have facilitated greater accuracy of EBV and lower correlations of EBV between relatives [12], particularly in younger stock that have not had any performance measurements yet, or if the trait is sex-limited (i.e. milk production), is hard-to-measure (i.e. carcass traits), or has a low heritability (i.e. reproduction).

Recently, some studies have assessed some of the synergies that may exist between the use of reproductive technologies and genomic selection. Using stochastic simulation, Pederson et al. [13] found that genomic selection and MOET on 50 % of nucleus dairy breeding cows resulted in a 23 % increase in genetic gain with no significant increase in inbreeding. Pryce et al. [14] used a deterministic model for a dairy breeding program to show a 98 % increase in genetic gain per year when exclusively using JIVET and genomic selection compared to a progeny test program, but with an increase of inbreeding rate by 65 %. It is important for practical breeding programs to consider numbers of female selection candidates consigned to MOET and JIVET since both are expensive and labor intensive procedures. It is also important to investigate strategies to slow rates of inbreeding which can be accelerated by these technologies.

It is clear from previous studies [3–8, 10, 13, 14] that increasing selection intensity for females in breeding programs can significantly increase rates of inbreeding. Optimal contribution selection (OCS) is a method that balances longer-term genetic merit and genetic diversity in breeding populations. OCS can determine the optimal levels of the genetic contributions of selection candidates but these are often limited by physiological parameters under natural mating. OCS could, however, be an effective tool to assign female reproductive technologies to individual females in breeding programs, while also assigning optimal contributions to males. For a fair comparison and a realistic assessment of the value of reproductive technologies and GS and their potential synergies, comparisons of breeding programs should be done at similar inbreeding rates.

The method developed by Wray and Goddard [15] that applies multiple penalties to co-ancestry while allocating optimal genetic contributions from selection candidates allows the entire selection space between genetic gain and inbreeding to be explored rather than targeting a specific point as demonstrated by Meuwissen [16]. Wray and Goddard’s [15] method is useful to investigate the optimal balance between genetic gain and inbreeding. Since OCS allows genetic diversity to be maintained and genetic contributions to be optimized, the synergies that exist between GS and reproductive technologies can be further explored via simulation of breeding programs.

The objective of this study was to assess the value of using female reproduction technologies combined with GS and compare their benefits in beef and dairy cattle and sheep breeding programs, when the aim is to increase rates of genetic gain while keeping inbreeding at sustainable levels. We applied OCS with various penalties on inbreeding and optimized the number of females to be used in MOET or JIVET with regard to genetic gain and inbreeding.

## Methods

### Simulation

Stochastic simulation was used to model a number of closed nucleus breeding schemes. The number of progeny born each year in a closed nucleus was 250, 250 and 600 for sheep, beef and dairy cattle, respectively. For each scenario, we generated a base population of unrelated animals, and subsequently established a 20-year breeding program with overlapping generations. There was an annual random death rate of 10 %. Phenotypes and selection were for a single trait for each breeding program. The genetic values for the base individuals were simulated using a polygenic model as:

$$a_{i } = z\sigma_{a} ,$$

where *z* is a random variable drawn from a standard normal distribution and *σ*_*a*_ is the genetic standard deviation of the trait. Breeding values for the subsequent generations were obtained using:

$$a_{i} = ((a_{si} + a_{di} )/2) + MS_{i} ,$$

where *a*_*si*_ and *a*_*di*_ are the true breeding values of the sire and dam of animal *i*, respectively, and *MS*_*i*_ is the Mendelian sampling effect for individual *i,* which was simulated as:

$$MS_{i} = z \cdot \sigma_{a} \sqrt {\left( {.5(1 - \overline{F}_{i} )} \right)} ,$$

where $\mathop {\overline{{F_{i} }} }\limits^{{}}$ is the average inbreeding coefficient of the parents of individual *i*. Phenotypes were then simulated by adding a random error term to *a*_*i*_, such that each trait had a heritability of 0.3 in the base population.

We simulated a number of single-trait scenarios across species that differed in gestation length and whether trait measurement was before or after selection or sex-limited. Phenotypic information was available on selection candidates when the trait could be measured: (1) on both sexes within 6 months of age in sheep (e.g. weight and scan traits); (2) at 2 years of age in sheep on both sexes (e.g. adult wool production); (3) within the first year in beef cattle on both sexes (e.g. weaning and yearling weight); (4) at 19 months of age, after sexual maturity, in beef cattle on both sexes (e.g. 600 day live weight); (5) a sex-limited trait measured on females at 27 months of age (e.g. milk production).

### Eligibility and selection of males

#### Sheep

Rams were eligible for selection at 7 months of age and again at 19 months of age. Thereafter, they were culled if not selected at 19 months of age.

#### Beef cattle

Bulls were eligible for selection at 15 and 27 months of age. Thereafter, they were culled if not selected at 27 months of age.

#### Dairy cattle

In breeding programs without GS, bulls were eligible for pre-selection for progeny testing at 15 months of age. Young bulls were selected for progeny testing by conducting the OCS procedure with the 75 highest allocations of contributions selected. By the age of 4.5 years, each bull had 50 daughters (in external herds) with a lactation record. The progeny tested bulls were first eligible for mating in the closed nucleus at 4 years of age and all were kept as selection candidates at 5-years of age. Any bull not selected at 6 years of age or older was culled and all bulls were culled at 9 years of age. Similar to Pryce et al. [14], breeding programs that incorporated GS made bulls eligible for mating at 15 months old. After GS, at 15 months of age, OCS of the top 75 males based on optimal contribution was performed and they were then used in a progeny-testing program with 50 recorded daughters, similar to the breeding program without GS. In breeding programs where GS was used, all bulls that were selected for progeny-testing were eligible for selection every year up to 5 years of age.

### Genomic selection

Some breeding programs had a scenario where genomic selection was used for all animals born in the nucleus. Genomic information was modeled following the method of Dekkers [17], which simulates a genomic estimated breeding value (GEBV) as a correlated trait with a heritability of 0.99 and a correlation r to the measured trait, where r is the accuracy of the GEBV. The accuracy of the GEBV was assumed to be 0.5 for sheep [18], 0.5 for beef cattle [19] and 0.7 for dairy cattle [20]. All animals received a GEBV at birth and the GEBV was combined with phenotypic information when calculating breeding values.

### Estimation of breeding values

Each year, EBV were estimated using best linear unbiased prediction (BLUP). The model assumed used was:

$${\mathbf{y}} = {\varvec{1_n}} \mu + {\mathbf{Za}} + {\mathbf{e}},$$

where **y** is a vector of phenotypes, *µ* is the mean, ${\varvec{1_n}}$ is a vector of 1s, **Z** is a design matrix allocating records to breeding values, **a** is a vector of breeding values with ${\text{var}}\left( {\mathbf{a}} \right) = {\mathbf{A}}\sigma_{a}^{2}$ where **A** is the numerator relationship matrix and $\sigma_{a}^{2}$ is the additive genetic variance, **e** is a random effect. All data from the base population onwards were used for genetic evaluation, including data on the progeny of sires that were progeny-tested.

### Optimal contribution selection

Optimal contribution selection was implemented using the approach of Wray and Goddard’s [15], balancing the average genetic merit (M) of selected individuals with the average co-ancestry among selected individuals (C), both weighted by their contributions to the next generation. Average genetic merit of the selected individuals was evaluated as:

$${\mathbf{M}} = {\mathbf{x}}^{{\prime }} \mathop {{\hat{\mathbf{a}}}}\limits^{{}} ,$$

where $\mathop {{\hat{\mathbf{a}}}}\limits^{{}}$ is a vector of EBV and **x** is a vector of genetic contributions of selection candidate animals, with values in **x** summing to 0.5 for each of the sexes. The **x** vector contributions for females were capped at a maximum of the equivalent of four offspring per female when MOET or JIVET was used and the equivalent of 1 for the scenarios with only artificial insemination or natural mating (AI/N). After contributions were assigned to females, the number of required matings could be determined for males. The maximum number of matings for a single male was then capped at the total number of female matings. Price and Storn’s [21] evolutionary algorithm was used to find optimal solutions for M + C. Once female and male candidates were assigned a mating, they were placed on a mating list. Matings were assigned at random and females were given a chance of a random number of live progeny according to a distribution shown in Table 1. One mating could result in multiple progeny via twins with AI/N, and via embryos implanted in recipients in MOET and JIVET programs.

**Table 1:** Probability of producing a certain number of live progeny per female per mating for artificial insemination (AI) or natural mating (AI/N), MOET and JIVET in different species

Progeny number/program	AI/N12^a^	AI/N^a^	AI/N^c^	AI^d^	MOET^b,e^	JIVET^b^
Species	Sheep	Sheep	Beef	Dairy	All	All
0	0.40	0.10	0.07	0.15	0.10	0.25
1	0.58	0.70	0.92	0.85	0.05	0.05
2	0.02	0.20	0.01	0.00	0.05	0.18
3	0.00	0.00	0.00	0.00	0.15	0.18
4	0.00	0.00	0.00	0.00	0.25	0.10
5	0.00	0.00	0.00	0.00	0.15	0.10
6	0.00	0.00	0.00	0.00	0.13	0.07
7	0.00	0.00	0.00	0.00	0.07	0.04
8	0.00	0.00	0.00	0.00	0.05	0.03
Average number of progeny	0.62	1.1	0.94	0.85	4.02	8.37^f^
Range of progeny possible	0–2	0–2	0–2	0–1	0–8	0–24

*AI/N7* probability for a ewe at 12 months of age of having a lamb

^a^[22]

^b^[23]

^c^[24]

^d^[25]

^e^[26]

^f^Predicted average of total progeny of three JIVET matings

Inbreeding rates were managed by penalizing the average co-ancestry among selected animals, which was computed as:

$${\text{C }} = \, \lambda {\mathbf{x}}^{{\prime }} {\mathbf{Ax}},$$

where **A** is the (n × n) relationship matrix among all candidates based on pedigree information and λ is a penalty that can be set to result in different rates of inbreeding. Price and Storn’s [21] evolutionary algorithm was then used to find optimal contributions as in the vector **x** by maximizing M + C for a given value of λ. These λ values evaluated included (0, −15, −25, −35, −50, −75, −100, −250, −1000, −9999). The values of λ were selected to explore the entire selection space of inbreeding vs genetic gain, which when combined, would form a frontier of possible outcomes. Smaller penalties will result in higher levels of co-ancestry among selected candidates, with potentially higher rates of genetic gain and harsh penalties will result in higher genetic diversity with lower rates of genetic gain.

### Breeding programs

Three breeding program structures were simulated that differed in the use of reproductive technologies. The first breeding program used only natural mating and/or AI and was simulated as a control. The second breeding program examined the effect of adding MOET to the breeding program, and the third examined the effect of MOET and JIVET combined. The number of offspring that each dam produced under the various reproductive technologies was randomly assigned from a range of offspring based on previous studies [22–26] (Table 1). Females were only offered one chance of being allocated a reproductive technology in each selection round. If no offspring were born, the female was carried as a dry animal to the next round of selection.

#### Breeding program 1: artificial insemination/natural mating (AI/N)

Ewes were eligible for selection at 7 and 19 months of age. Heifers and cows’ were eligible for selection at 15 and 27 months of age in the beef and dairy breeding programs. Ewes and cows were culled if not selected at 19 and 27 months of age, respectively.

#### Breeding program 2: AI/N + MOET

Eligibility of females for AI/N or MOET was the same as in the AI/N breeding program because MOET can only be performed on sexually mature females, including young ewes that are just entering puberty [27]. Females that were selected to contribute four offspring based on OCS were assigned to MOET. Females selected to contribute between one and three offspring were assigned to AI/N. Eligibility and culling of females were the same as in the AI/N breeding programs. In all MOET programs, dams were given the chance of having 0 to 8 offspring, with an average of 4 (Table 1). Females were only assigned one male mate for each AI/N or MOET breeding.

#### Breeding program 3: AI/N + MOET + JIVET

The third breeding program added the possibility for juvenile females to be candidates for selection through the use of JIVET. Only juvenile ewes and heifers were eligible for JIVET and they were not eligible for AI/N or MOET in either species. Juvenile females that were selected in OCS to four offspring were assigned JIVET matings. Sexually mature females that were selected to contribute three offspring were assigned to MOET, females selected to contribute less than three offspring were assigned an AI mating, and females that were selected to contribute less than one offspring were not used.

Due to the possibility of using multiple sires in JIVET, three separate matings were assigned to females for JIVET in the same mating period, whereas AI/N and MOET females only received one mating. Selected juvenile females could have between 0 and 8 offspring for each of the three matings, with an average of 2.8 live offspring per mating (Table 1).

Ewes were 1 month old at selection for JIVET. Sheep breeding programs required a mid-year JIVET mating since the timing of JIVET-on-JIVET programs do not fit into annual breeding programs. During the mid-year mating, all ewe selection candidates (including mature ewes) were entered into OCS but only JIVET allocations were executed despite AI and MOET matings being allocated to the mature ewes. As these matings allocated to mature ewes did not yield any progeny, the following round of selection ignored those allocated matings. OCS took into account if ewes had contributed 6 months earlier. In beef and dairy breeding programs, heifers were 3 months old when assigned to JIVET and did not require a mid-year mating like sheep as cattle have a nine month gestation period.

### Comparison of breeding programs

Each breeding program was run for 20 years and replicated 75 times. Breeding programs were compared based on average annual genetic gains and average annual rates of inbreeding for years 6 to 20, i.e. after the Bulmer equilibrium [28] was reached. Inbreeding was calculated annually as the average inbreeding coefficient of the entire drop of animals born that year using pedigree information. The average annual inbreeding to be displayed in the figures was calculated as the inbreeding increase per year averaged from years 6 to 20 of the simulation. Inbreeding per generation was also calculated due to overlapping generations existing in the simulations. Inbreeding per generation was calculated by matching the average inbreeding coefficient over the last 10 years with the number of generations in *n* years where:

$$NGens = \left( {\frac{n}{L}} \right) ,$$

where *n* is the number of breeding years and *L* is the average generation interval over the final 15 years of the simulated breeding program. Generation interval for each individual was calculated as average age of parents at age of birth. The annual generation interval was calculated as the average generation interval of the entire drop born each year.

## Results

### Differences between breeding programs without genomic selection

**Fig. 1:**
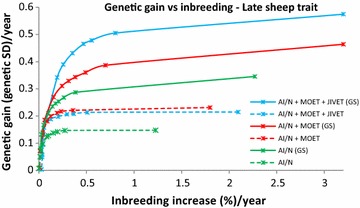
Average annual genetic gain (genetic standard deviations) and rate of inbreeding (%) with selection on a late measured trait in sheep using reproductive technologies with and without genomic selection. Data points represent different penalties on coancestry, with no penalty on the far *right* and a high penalty on the far *left*

**Fig. 2:**
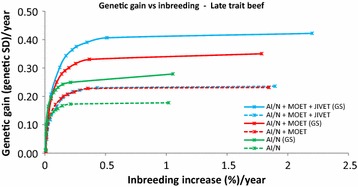
Average annual genetic gain (genetic standard deviations) and rate of inbreeding (%) with selection on a late measured trait in beef using reproductive technologies with and without genomic selection. Data points represent different penalties on coancestry, with no penalty on the far *right* and a high penalty on the far *left*

**Fig. 3:**
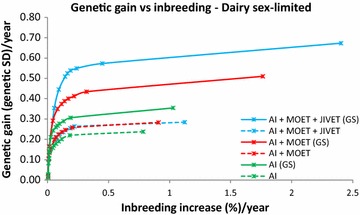
Average annual genetic gain (genetic standard deviations) and rate of inbreeding (%) in dairy cattle using reproductive technologies with and without genomic selection. Data points represent different penalties on coancestry, with no penalty on the far *right* and a high penalty on the far *left*

**Fig. 4:**
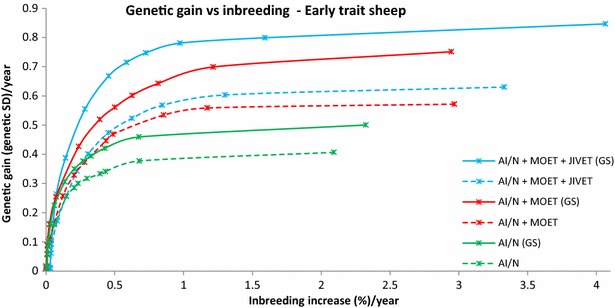
Average annual genetic gain (genetic standard deviations) and rate of inbreeding (%) with selection on an early measured trait in sheep using reproductive technologies with and without genomic selection. Data points represent different penalties on coancestry, with no penalty on the far *right* and a high penalty on the far *left*

**Fig. 5:**
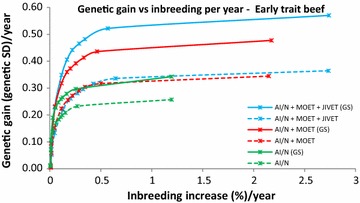
Average annual genetic gain (genetic standard deviations) and rate of inbreeding (%) with selection on an early measured trait in beef using reproductive technologies with and without genomic selection. Data points represent different penalties on coancestry, with no penalty on the far *right* and a high penalty on the far *left*

At moderate (1 % per generation) to high (>1.5 % per generation) levels of inbreeding MOET outperformed AI in all cases by 25 to 50 %. At lower inbreeding levels, MOET provided up to 17 to 65 % more genetic gain than AI/N, particularly for late (Figs. 1, 2) and sex-limited traits (Fig. 3). When inbreeding was heavily penalized, there was little genetic gain or inbreeding. In the early measured sheep trait, JIVET yielded 4 to 10 % more genetic gain than MOET (Fig. 4). However, for a late sheep trait and for both beef traits and a sex-limited dairy trait, there was no significant difference in genetic gain between the JIVET and MOET breeding programs (Figs. 1, 2, 3, 5) although some matings were allocated to JIVET (Table 2). As the penalty on inbreeding increased, generation intervals increased and genetic gain decreased (Table 3). It should also be noted that “unpenalized” inbreeding levels in Tables 3 and 4 are of the results of setting λ to zero which are similar to results from truncation selection.

**Table 2:** Proportion of selected females assigned each type of mating in each breeding program when rate of inbreeding is 1 % (±0.05 %) per generation for sheep, beef and dairy

	AI/N	MOET	JIVET	Nb of females selected^a^	Nb of males used^a^	Nb of females per male
Early trait sheep						
AI/N + MOET + JIVET (GS)	0.37	0.24	0.39	55	23	2.4
AI/N + MOET + JIVET	0.42	0.37	0.21	65	24	2.7
AI/N + MOET (GS)	0.41	0.59		95	23	4.0
AI/N + MOET	0.5	0.5		110	23	4.8
AI/N (GS)	1			243	28	8.7
AI/N	1			243	31	8.0
Late trait sheep						
AI/N + MOET + JIVET (GS)	0.36	0.18	0.46	51	28	1.8
AI/N + MOET + JIVET	0.42	0.29	0.29	59	27	2.2
AI/N + MOET (GS)	0.34	0.66		110	27	4.1
AI/N + MOET	0.45	0.55		110	29	3.8
AI/N (GS)	1.00			245	33	7.4
AI/N	1.00			244	32	7.7
Early trait beef						
AI/N + MOET + JIVET (GS)	0.24	0.32	0.44	47	19	2.5
AI/N + MOET + JIVET	0.26	0.35	0.39	57	20	2.9
AI/N + MOET (GS)	0.34	0.66		98	18	5.4
AI/N + MOET	0.34	0.66		100	19	5.3
AI/N (GS)	1.00			276	14	19.7
AI/N	1.00			277	14	19.8
Late trait beef						
AI/N + MOET + JIVET (GS)	0.25	0.34	0.41	51	14	3.6
AI/N + MOET + JIVET	0.26	0.40	0.34	57	15	4.1
AI/N + MOET (GS)	0.32	0.68		97	15	7.7
AI/N + MOET	0.35	0.65		101	16	5.0
AI/N (GS)	1.00			277	15	18.1
AI/N	1.00			279	16	17.2
Dairy						
AI + MOET + JIVET (GS)	0.38	0.28	0.34	143	39	3.7
AI + MOET + JIVET	0.47	0.35	0.18	167	41	4.1
AI + MOET (GS)	0.47	0.53		265	40	6.7
AI + MOET	0.44	0.56		263	34	7.7
AI (GS)	1.00			646	59	11.0
AI	1.00			652	41	15.8

^a^All standard errors of means (SEM) for total numbers of ewes, cows and males were less than 1.2, 2.7 and 0.18, respectively

**Table 3:** Average generation interval (*L*) and annual genetic gain (ΔG in *σ*_*a*_ units) across 75 replicates at various rates of inbreeding (±0.05 %) per generation for sheep, beef and dairy traits

Inbreeding rate (%/generation)	0.5		1.0		Unpenalized	
	L	ΔG	L	ΔG	L	ΔG
Early trait sheep						
AI/N + MOET + JIVET (GS)	1.06	0.67	1.01	0.77	0.92	0.85
AI/N + MOET (GS)	1.73	0.43	1.39	0.63	1.23	0.75
AI/N (GS)	2.04	0.39	1.94	0.44	1.84	0.50
AI/N + MOET + JIVET	1.30	0.40	1.19	0.59	0.98	0.63
AI/N + MOET	1.73	0.37	1.48	0.51	1.27	0.57
AI/N	2.16	0.32	2.02	0.34	1.90	0.41
Late trait sheep						
AI/N + MOET + JIVET (GS)	1.07	0.46	1.03	0.51	1.01	0.58
AI/N + MOET (GS)	1.64	0.34	1.43	0.39	1.41	0.46
AI/N (GS)	2.25	0.25	2.08	0.29	1.96	0.35
AI/N + MOET + JIVET	1.49	0.20	1.42	0.21	1.32	0.22
AI/N + MOET	3.20	0.21	3.12	0.22	3.03	0.23
AI/N	3.24	0.14	3.13	0.15	3.06	0.15
Early trait beef						
AI/N + MOET + JIVET (GS)	2.28	0.41	2.1	0.49	1.99	0.57
AI/N + MOET (GS)	2.93	0.36	2.71	0.41	2.59	0.48
AI/N (GS)	3.92	0.27	3.69	0.29	3.70	0.34
AI/N + MOET + JIVET	2.63	0.25	2.27	0.31	2.19	0.36
AI/N + MOET	3.10	0.26	2.9	0.3	2.68	0.34
AI/N	4.04	0.20	3.92	0.21	3.86	0.26
Late trait beef						
AI/N + MOET + JIVET (GS)	2.38	0.36	2.24	0.41	2.14	0.42
AI/N + MOET (GS)	3.10	0.29	2.97	0.33	2.86	0.35
AI/N (GS)	4.08	0.22	3.93	0.26	3.92	0.28
AI/N + MOET + JIVET	2.72	0.21	2.58	0.23	2.51	0.24
AI/N + MOET	3.36	0.21	3.19	0.23	3.00	0.23
AI/N	4.18	0.17	4.15	0.17	4.03	0.18
Dairy						
AI + MOET + JIVET (GS)	1.92	0.54	1.86	0.58	1.79	0.67
AI + MOET (GS)	2.66	0.40	2.58	0.44	2.46	0.51
AI (GS)	3.55	0.28	3.41	0.32	3.35	0.35
AI + MOET + JIVET	4.09	0.24	3.97	0.26	3.91	0.28
AI + MOET	4.62	0.24	4.52	0.26	4.47	0.28
AI	5.55	0.18	5.39	0.21	5.28	0.24

All standard errors of means (SEM) were less than 0.11 and less than 0.003 for all breeding programs for generation interval and genetic gain, respectively

**Table 4:** Increase (%) in rate of genetic gain per year of breeding programs using MOET and JIVET compared to the AI/N breeding program (Repro) and increase (%) in rate of genetic gain per year from the use of genomic selection (GS) on each breeding program at various rates of inbreeding (±0.05 %) per generation for sheep, beef and dairy traits

Inbreeding rate (%/gen)	0.5		1.0		Unpenalized	
	Repro	GS	Repro	GS	Repro	GS
Early trait sheep						
AI/N + MOET + JIVET (GS)	72	68	75	31	70	35
AI/N + MOET (GS)	10	16	43	24	50	32
AI/N (GS)		22		29		22
AI/N + MOET + JIVET	25		74		54	
AI/N + MOET	16		50		39	
Late trait sheep						
AI/N + MOET + JIVET (GS)	84	130	76	143	66	163
AI/N + MOET (GS)	36	62	34	77	31	100
AI/N (GS)		79		93		133
AI/N + MOET + JIVET	43		40		47	
AI/N + MOET	50		47		53	
Early trait beef						
AI/N + MOET + JIVET (GS)	52	64	69	58	68	58
AI/N + MOET (GS)	33	38	41	37	41	41
AI/N (GS)		35		38		31
AI/N + MOET + JIVET	25		48		38	
AI/N + MOET	30		43		31	
Late trait beef						
AI/N + MOET + JIVET (GS)	64	71	58	78	50	75
AI/N + MOET (GS)	32	38	27	43	25	52
AI/N (GS)		29		53		56
AI/N + MOET + JIVET	24		35		33	
AI/N + MOET	24		35		28	
Dairy						
AI + MOET + JIVET (GS)	93	125	81	123	91	139
AI + MOET (GS)	43	67	38	69	46	82
AI (GS)		56		52		46
AI + MOET + JIVET	33		24		17	
AI + MOET	33		24		17	

### Differences between breeding programs with genomic selection

When GS was implemented in breeding programs, programs using JIVET yielded the largest genetic gain, followed by programs using MOET for all species and traits (Table 3; Figs. 1, 2, 3, 4, 5). The largest differences in genetic gain between JIVET and MOET breeding programs were observed for late traits in sheep and beef cattle and for a sex-limited trait in dairy cattle, with differences ranging from 24 to 32 % of genetic gain with MOET at moderate inbreeding levels. Differences in annual genetic gain between MOET and AI/N were largest for early measured traits, ranging from 41 to 43 % of genetic gain with AI (Tables 3, 4). The use of both JIVET and MOET decreased generation intervals, with programs using JIVET displaying the most significant decrease for all species and traits (Table 3).

### Impact of genomic selection on breeding programs

The use of GS decreased generation intervals and increased genetic gain across all species and traits (Table 3). Genomic selection increased genetic gain by 37 to 143 % for breeding programs using JIVET, 23 to 77 % for breeding programs using MOET and 23 to 93% for AI/N breeding programs (Table 4). These gains were proportionally higher for late measured traits in sheep (Fig. 1), beef cattle (Fig. 2) and dairy cattle (Fig. 3). The use of GS in early measured traits for sheep (Fig. 4) and beef cattle (Fig. 5) still produced 20 to 40 % more genetic gain when combined with reproductive technologies.

When inbreeding was not heavily penalized (>0.5 % inbreeding per generation), the allocation of reproductive technologies increased with the use of GS. When comparing breeding programs with and without GS, there were more JIVET matings with GS at the expense of MOET matings, and this re-allocation was more pronounced for late measured traits and in dairy cattle (Table 3).

## Discussion

### Breeding programs

Our results show that, as in prior studies, using female reproductive technologies increases genetic gain when compared to traditional breeding programs. Moreover, we found that the use of female reproductive technologies in combination with the implementation of genomic selection increased rates of genetic gain even more. However, we also observed in all scenarios that if inbreeding was not penalized, large inbreeding coefficients could be accumulated over time. This study demonstrates the benefits of optimizing the allocation of females to reproductive technologies through the use of optimal contribution selection, which resulted in increased rates of genetic gain under sustainable rates of inbreeding. Combining MOET with GS yielded 25 to 50 % more genetic gain compared to AI and combining JIVET with GS increased genetic gain up to 143 % without increasing the rate of inbreeding.

Genomic selection resulted in clear benefits between breeding strategies for all traits, species and breeding programs using different technologies. When comparing breeding programs with and without GS, increases in genetic gains from the use of GS ranged from 33 to 143 % for JIVET programs, from 37 to 77 % for MOET programs and from 29 to 93 % for AI/N programs (Tables 3, 4). When comparing programs that used GS, JIVET led to 15 to 43 % greater genetic gain than MOET breeding programs. We also observed a 38 to 76 % greater genetic gain for MOET breeding programs compared to AI/N breeding programs when GS was implemented in both breeding programs. These gains of MOET over AI and JIVET over MOET were due to greater accuracies of EBV at selection of young selection candidates and lower correlations between the EBV of siblings. Genomic selection allows an increase of selection accuracy at the age of first selection and this increase is greatest for late and sex-limited traits. Genomic information increases accuracy of EBV by explaining within-family variance due to Mendelian sampling [12], which is important when selecting animals early because without GS there is little information about this component. Moreover, the Bulmer effect reduces the between-family variance and, therefore, contributes less to future genetic gains than the within-family variance [29]. Dekkers [17] concluded that highly accurate (0.8) GEBV could increase genetic gain by 21 and 81 % over a traditional phenotypic selection program for traits with a heritability of 0.3 and 0.1, respectively. These extra gains from GS depend on whether traits have been measured on selection candidates at the time of first selection. In dairy cattle, we used accurate GEBV for GS (0.7) and observed that GS increased rates of genetic gain in traditional AI programs by 52 % (Tables 3, 4). Using GS in sheep and beef cattle programs, with a lower accuracy of GEBV (0.5), increased rates of genetic gain by 29 to 93 %, depending on species and when the trait was measured (Tables 3, 4). Genomic predictions in beef cattle and sheep are typically less accurate than in dairy cattle due to the extensive and multi-breed aspect of these industries, which makes it more difficult to generate training populations that sustain high accuracies. Studies in sheep that assumed maximum accuracies of GEBV of 0.5 showed that GS increased genetic gain by 21 % in terminal sire breeds and up to 39 % in fine wool Merino sheep [30].

### Optimal contribution selection

While many previous studies have evaluated the short-term benefits of reproductive technologies, they did not all explore how the rate of inbreeding can be minimized or reduced. Wray and Goddard [15] developed a selection principle to manage rates of inbreeding through a penalty method. Other studies implement OCS by maximizing genetic gain at a specific rate of inbreeding [16, 31, 32]. Together, these methods have demonstrated that maintaining a high level of genetic gain at set rates of inbreeding can be achieved. We used a range of inbreeding penalties to create ‘frontiers’ of genetic gain versus inbreeding, thereby clearly showing the balance that can be achieved (Figs. 1, 2, 3, 4, 5), rather than targeting specific inbreeding rates. We were then able to choose specific inbreeding rates per generation to compare scenarios. While genetic gain somewhat plateaued when the rate of inbreeding increased to 1 % per generation (Table 3), managers of breeding programs must decide which point on the frontier suits their breeding objective.

Our study is unique in that it uses OCS to optimize genetic gain with inbreeding in livestock breeding programs that use female reproductive technologies. It also optimally assigned reproductive technologies according to how much each mating contributes to the objective function of maximizing gain while penalizing inbreeding. Table 2 demonstrates the proportion of females that were allocated to different reproductive technologies. We observed that, with the added accuracy that is provided by GS, the proportions of MOET allocations did not change in AI + MOET breeding programs. However we observed that with the use of GS in AI + MOET + JIVET breeding programs increased JIVET allocations by up to 86, 20 and 88 % and decreased MOET allocations by up to 38, 15 and 20 % for sheep, beef cattle and dairy cattle breeding programs, respectively. In conjunction with a shift in the selection of more JIVET candidates, we observed a decrease in generation intervals (Table 3). We also observed that the number of males selected each year differed only slightly between programs (Table 2). It should be noted that males are generally used below their maximum reproductive capacity. The use of males below their natural or artificial insemination capabilities was needed to maintain diversity without sacrificing too much genetic gain. The concept of reducing inbreeding while implementing reproductive technologies by using more sires under their reproductive capacity has been described previously by Kinghorn [33], Arendonk and Bijma [34], Pryce et al. [14] and Lillehammer et al. [35] in dairy, and by Brash et al. [8] in sheep breeding schemes.

### Comparing with previous studies

Our study estimated that the benefit of MOET in sheep breeding programs without using GS, was up to 50 % while maintaining the rate of inbreeding at 1 % per generation. Previous studies [7, 8] using MOET in sheep breeding programs also predicted such gains but without restricting inbreeding rates. Our beef MOET breeding programs achieved 35 to 43 % more genetic gain than AI programs without GS which was less than previous studies, which reported extra gains of 67 to 138 % [7, 9, 10], but we achieved these gains at the same rate of inbreeding per generation, while rates of inbreeding increased by 87 to 300 % in the aforementioned studies. Use of MOET in our dairy breeding program resulted in 24 % extra genetic gain without increasing the rate of inbreeding. This is similar to the values reported by Kinghorn [33] and Leitch et al. [3], who estimated similar gains in dairy cattle nucleus breeding programs that implemented MOET compared to traditional AI nucleus breeding programs but with larger increases in rates of inbreeding (1.7 to 4.2 % increase per year). Without GS, all traits and species benefited from the use of MOET. However, except for the early measured trait in sheep, which yielded 4 to 10 % greater annual genetic gain, the use of JIVET without GS did not yield extra genetic gain compared to AI + MOET programs in late trait sheep, beef and dairy cattle breeding programs, although some JIVET matings were allocated to juveniles (Table 2). The lower allocation of JIVET matings is likely due to the low accuracy of EBV and the high correlation between EBV of full siblings at a young age. This demonstrates that, even without accounting for the additional cost of JIVET, this technology may not give an advantage to breeding programs when using optimal contribution selection across age classes, at least without GS.

Previous studies have also evaluated breeding programs that implement female reproductive technologies with GS in comparison to traditional programs. Pryce et al. [14] deterministically modeled a closed nucleus of Holstein cattle in which 300 juvenile females that were submitted to JIVET were mated only to genomically selected sires and compared the rates of genetic gain and inbreeding to a progeny-testing AI breeding program. The JIVET program resulted in a 131 % increase in genetic gain and a 7 % decrease in rate of inbreeding, which they obtained by increasing the number of sires of bulls and cows. Their breeding program resulted in 0.59 genetic standard deviations of genetic gain per year at a 0.17 % rate of inbreeding per year. The most comparable scenario in our study is the AI + MOET + JIVET (GS) program vs. AI. With a rate of inbreeding of 0.17 % per year for both programs, we observed that AI + MOET + JIVET (GS) resulted in a 145 % greater genetic gain per year than the AI breeding program, with gains of 0.54 and 0.22 genetic standard deviations per year, respectively (Fig. 5). The genetic gain that we obtained in the dairy cattle AI + MOET + JIVET (GS) breeding program was not quite as large as that in Pryce et al. [14]. This could be due to our stochastic simulation also using MOET and AI technologies which resulted in higher generation intervals and lower selection intensities compared to Pryce et al.’s [14] 100 % JIVET each year.

### Rates of inbreeding

Breeding programs resulting in high rates of inbreeding should be avoided due to the likelihood of declines in fitness [36, 37] and homozygous recessive genotypes that are lethal [38]. However, if inbreeding is penalized too heavily, genetic diversity is maintained but with low rates of genetic gain (Figs. 1, 2, 3, 4, 5). Therefore, a compromise must be made to achieve desirable amounts of genetic gain without increasing inbreeding too much. Figures 1, 2, 3, 4 and 5 show rates of inbreeding per year, but past studies suggest using inbreeding rate per generation as a metric as it can be used to compare different breeding programs with different generation intervals. Bijma [39] suggested restricting rates of inbreeding to 1 % per generation, which was supported by Buch et al. [40].This desired rate of inbreeding will differ between producers due to varying concerns about the importance of inbreeding, the size of the nucleus, the ability to import unrelated animals into the nucleus, the effective population size of the breed as a whole, and the incidence of recessive homozygous genetic diseases [41].

### Simulation restrictions

In this study, GEBV were simulated as a correlated trait with a heritability of 1 and a genetic correlation with the trait equal to the accuracy of the GEBV [24]. Genomic selection can also be simulated by simulating quantitative trait loci (QTL) and genetic markers. However, because we did not simulate genotypes, predicting the linkage disequilibrium (LD) patterns across generations [42] between markers and QTL was not addressed in our simulations. Family sizes that increase in a nucleus over generations through the use of MOET and JIVET could slightly increase the accuracy of the individual GEBV of those family members [43]. However, predicting the changes in gene frequencies of the nucleus population using an infinitesimal model could be difficult to determine over a period longer than three generations [44]. An alternative method that simulates the actual marker genotypes could be used to capture the change in LD structure over time due to selection [45]. Some studies [46–48] have studied the efficacy of genomic selection when performed over many generations, and where only a few QTL control genetic variation. They suggest that the effects of markers do not change but the proportion of genetic variance explained by them will decline, which means that the effects of genomic selection may be over-estimated in our simulations. However, it has been suggested that hundreds or thousands of QTL may control variation for a given trait [49, 50]. Heffner et al. [51] demonstrated that if all QTL effects are small, as in an infinitesimal model, genomic selection could maintain accuracies over multiple generations due to the change in LD between QTL and markers being less important over a longer time period. Therefore, given both sides of the argument it is difficult to predict whether the method of Dekkers [17] over- or under-estimates genetic gains from genomic selection.

In our study, we used a pedigree-derived relationships matrix (**A**) to measure inbreeding and also during OCS. Some authors have shown that using a genomic relationship matrix (**G**) in OCS has some advantages over using **A** by achieving similar rates of genetic gain with slightly lower rates of inbreeding [52, 53]. This is achieved because **G** accounts for differences in the level of relatedness within families. Using **G** would however increase computing time for an already time-consuming simulation.

This study did not take the costs of technology into account. If they were, we expect that the use of reproductive technologies would decrease. In some cases, reproductive technologies were assigned although the additional genetic benefit was limited, in particular when inbreeding was highly penalized. Therefore, this study evaluated the maximum use and benefit in terms of genetic gain. Including costs will, however, give a more realistic picture of potential use. A cost benefit assessment will also focus more on how genetic gain is translated into actual monetary benefit for the breeder who carries the burden of the additional investment.

## Conclusions

Reproductive technologies combined with genomic selection can substantially enhance rates of genetic gain without compromising rates of inbreeding when optimal contribution selection is used with an incurred penalty on future co-ancestry. Without genomic selection, the use of JIVET in conjunction with MOET is not beneficial. These results suggest that synergies exist between the applications of reproductive technologies, genomic selection and optimal contribution selection. Optimal contribution selection was an effective tool in optimizing the allocations of combined reproductive technologies. The full exploration of the frontier between inbreeding vs. genetic gain production was facilitated by applying a range of penalties to co-ancestry of selection candidates.
